# Supplementary material for: Supervised Machine Learning Based Multi-Task Artificial Intelligence Classification of Retinopathies
Source: J Clin Med. 2019 Jun 18;8(6):872. doi: 10.3390/jcm8060872 (PMC6617139; doi:10.3390/jcm8060872)
Supplement: Supplementary file 1 [file jcm-08-00872-s001.pdf]

Supplementary materials

Univariate analysis

Table S1. Univariate analysis of individual OCTA features for control, DR and SCR cohorts.

	Control	DR	SCR	P values		
				Control vs. DR	Control vs. SCR	DR vs. SCR
**BVT (SCP)**	1.11 ± 0.07	1.18 ± 0.04	1.25 ± 0.08	0.016	<0.001	0.022
**BVC (μm)(SCP)**	17.47 ± 1.9	20.32 ± 3.8	21.43 ± 2.4	0.024	0.019	0.836
**VPI (SCP)**	10.26 ± 1.32	8.76 ± 2.60	8.43 ± 0.79	0.012	0.036	0.325
**BVD (%)**						
**C1 (SCP), 2mm**	40.16 ± 10.32	35.61 ± 8.11	36.08 ± 7.02	0.019	0.051	0.154
**C2 (SCP), 4mm**	47.53 ± 6.32	40.72 ± 4.17	44.24 ± 5.52	<0.001	<0.001	0.017
**C3 (SCP), 6mm**	47.96 ± 2.36	38.89 ± 3.31	42.84 ± 3.24	<0.001	<0.001	0.014
**C1 (DCP), 2mm**	42.72 ± 13.19	38.98 ± 6.09	40.28 ± 10.17	0.024	0.058	0.208
**C2 (DCP), 4mm**	49.16 ± 5.78	43.32 ± 7.09	42.20 ± 4.17	<0.001	0.011	0.095
**C3 (DCP), 6mm**	48.97 ± 3.18	41.75 ± 6.53	43.29 ± 4.30	<0.001	<0.001	0.005
**FAZ-A (SCP), mm^2**	0.30 ± 0.06	0.39 ± 0.04	0.43 ± 0.05	0.006	<0.001	0.008
**FAZ-A (DCP), mm^2**	0.39 ± 0.08	0.52 ± 0.06	0.54 ± 0.06	0.006	0.005	0.059
**FAZ-CI (SCP)**	1.14 ± 0.11	1.38 ± 0.13	1.47 ± 0.14	<0.001	<0.001	0.004
**FAZ-CI (DCP)**	1.18± 0.12	1. 41 ± 0.10	1.53 ± 0.13	<0.001	<0.001	0.002

[a]All values are presented as mean ± SD.

Table S2. Univariate analysis of individual OCTA features for NPDR stages.

	Mild NPDR	Moderate NPDR	Severe NPDR	P values		
				Mild vs. Moderate	Moderate vs. Severe	Severe vs. Mild
**BVT (SCP)**	1.14 ± 0.05	1.17 ± 0.06	1.23± 0.04	0.260	0.546	0.017
**BVC (μm)(SCP)**	18.06± 1.9	21.04 ± 2.2	21.86 ± 1.7	0.036	0.213	0.011
**VPI (SCP)**	9.94 ± 0.38	8.56 ± 0.15	7.79 ± 0.21	0.025	0.044	<0.001
**BVD (%)**						
**C1 (SCP), 2mm**	36.62± 9.03	36.01 ± 5.81	34.20 ± 9.38	0.019	0.154	0.041
**C2 (SCP), 4mm**	44.36 ± 6.72	40.81 ± 5.22	36.98 ± 6.50	<0.001	<0.001	0.005
**C3 (SCP), 6mm**	43.85 ± 3.38	38.95 ± 4.65	33.87 ± 4.24	<0.001	<0.001	0.014
**C1 (DCP), 2mm**	40.88 ± 10.37	38.78 ± 7.01	37.29 ± 8.16	0.042	0.658	0.018
**C2 (DCP), 4mm**	47.42 ± 4.83	43.39 ± 6.39	39.16 ± 7.25	<0.001	0.026	<0.001
**C3 (DCP), 6mm**	41.75 ± 11.08	42.32 ± 7.45	37.73 ± 5.29	<0.001	0.006	<0.001
**FAZ-A (SCP), mm^2**	0.33 ± 0.05	0.38 ± 0.07	0.46 ± 0.06	<0.001	<0.001	<0.001
**FAZ-A (DCP), mm^2**	0.46 ± 0.07	0.53 ± 0.12	0.58 ± 0.09	0.018	0.003	<0.001
**FAZ-CI (SCP)**	1.29 ± 0.14	1.38 ± 0.14	1.46 ± 0.18	<0.001	0.002	<0.001
**FAZ-CI (DCP)**	1.31 ± 0.21	1.42 ± 0.19	1.49 ± 0.17	<0.001	0.009	0.002

[a]All values are presented as mean ± SD

Table S3. Univariate analysis of individual OCTA features for SCR stages.

	**Mild SCR**	**Severe SCR**	**P values (Mild vs. severe)**
**BVT (SCP)**	1.22 ± 0.07	1.28± 0.05	<0.001
**BVC (μm)(SCP)**	18.82 ± 3.1	24.05 ± 2.6	0.385
**VPI (SCP)**	9.21 ± 0.26	9.64 ± 0.29	0.521
**BVD (%)**			
**C1 (SCP), 2mm**	36.99 ± 6.13	35.16 ± 8.08	0.163
**C2 (SCP), 4mm**	46.35 ± 4.53	42.13 ± 8.29	0.097
**C3 (SCP), 6mm**	46.85 ± 6.29	38.83 ± 3.23	0.018
**C1 (DCP), 2mm**	41.88 ± 10.85	38.68 ± 11.26	0.364
**C2 (DCP), 4mm**	47.06 ± 7.89	37.4 ± 8.36	0.073
**C3 (DCP), 6mm**	46.05 ± 6.25	40.5 ± 6.23	0.004
**FAZ-A (SCP), mm²**	0.41 ± 0.19	0..45 ± 0.12	<0.001
**FAZ-A (DCP), mm²**	0.52 ± 0.19	0.56 ± 0.17	<0.001
**FAZ-CI (SCP)**	1.45 ± 0.12	1.50 ± 0.15	<0.001
**FAZ-CI (DCP)**	1.50 ± 0.14	1.56 ± 0.16	0.002

[a]All values are presented as mean ± SD
